# A time-efficient workflow to purify a library of alanine mutants of surface-exposed residues of the domain III of the envelope protein of dengue virus serotype 1

**DOI:** 10.17912/micropub.biology.001012

**Published:** 2023-11-22

**Authors:** Dayron Martín Prieto, Alejandro M. Martín Dunn, Arturo Elías Llumbet, Luis Gabriel González-Lodeiro, Mónica Sarría Núñez, Cynthia González, Lázaro Betancourt, Gabriel Márquez Perera, Glay Chinea Santiago, Vivian Huerta Galindo

**Affiliations:** 1 Sericulture group, Estación Experimental de Pastos y Forrajes Indio Hatuey, Matanzas, Matanzas, Cuba; 2 System Biology, Centro de Ingeniería Genética y Biotecnología, Havana, Havana, Cuba; 3 Department of Biomedical Engineering, University Medical Center Groningen, Groningen, Groningen, Netherlands; 4 Department of Clinical research, Center of Molecular Immunology (Cuba), Havana, Havana, Cuba; 5 BMC - Biomedical Center, Lund University, Lund, Skåne, Sweden; 6 Department of Downstream development, Centro de Ingeniería Genética y Biotecnología, Havana, Havana, Cuba

## Abstract

Dengue complex is formed by four viral serotypes that cause the disease of the same name. Dengue is the arthropod-borne disease with the highest incidence worldwide. The envelope glycoprotein comprises three structural domains. The domain III (DIII) induces neutralizing antibodies and is involved in the interactions with soluble plasma factors from human host. Recombinant DIII proteins have been used as analytical tools for the characterization of virus-host interactions and have been evaluated as sub-unit vaccines. Here, we report a purification procedure of recombinant DIII protein and seventy-four alanine mutants refolding by size exclusion chromatography that allows obtaining highly homogeneous protein preparations and suitable for efficient purification and folding check. Four positions are identified that significantly affect either the protein expression or folding of recombinant DIIIE1, K310, G304, D330 and P332.

**Figure 1. Characterization of the heterologous expression in Escherichia coli and purification of DIIIE1wt and Ala-mutants:**
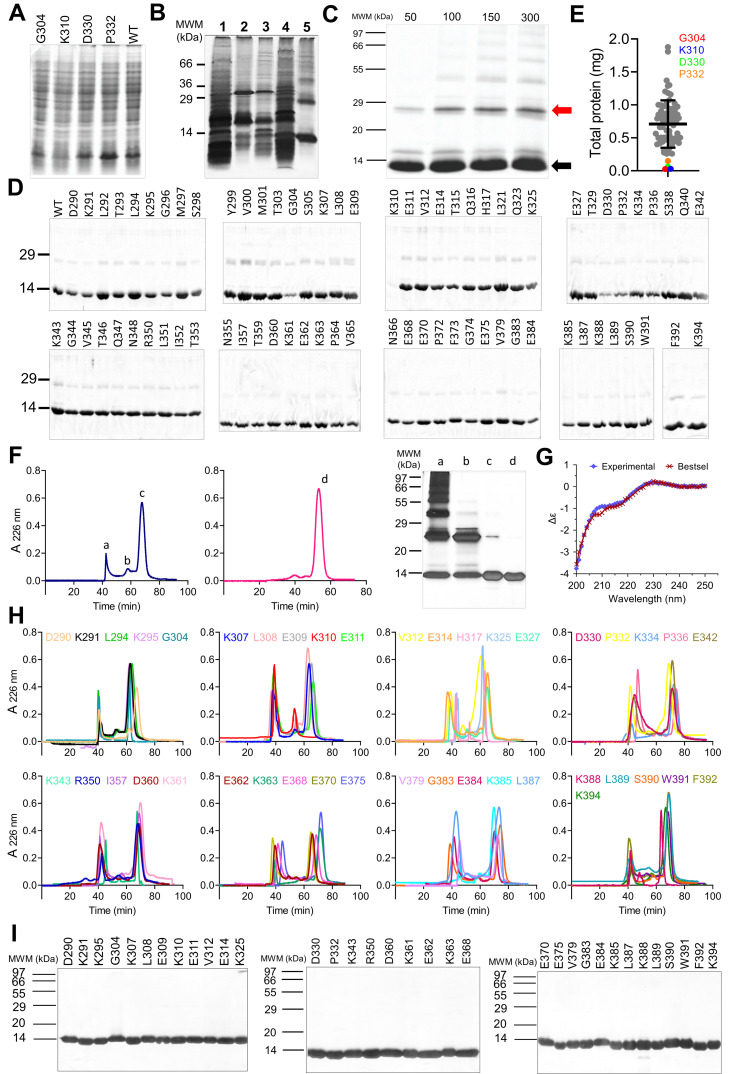
(A-D, F and I): Representative SDS-PAGE analysis. In C, F and I, samples were diluted in non-reducing sample buffer and protein bands were visualized by silver staining. The rest of SDS-PAGE analyses were performed under reducing conditions and gels were stained with coomassie blue. (A) Homogenates of E. coli cells transformed with the plasmids with constructs of DIIIE1wt and mutants, indicated on top of each lane. (B) Semipurification of inclusion bodies of DIIIE1wt. Lanes: 1. soluble fraction of the cell lysate; 2. Soluble fraction of the first wash using with TX1-100 1%; 3.soluble fraction of the second wash with urea 1M; 4. soluble fraction of the third wash with PBS 1X; 5. Inclusion bodies. (C) Fractions of batch elution of DIIIE1wt from Ni-NTA chromatography using increasing concentration of Imidazol (mmol/L), as indicated on top of each lane. The arrows indicate the migration corresponding to the protein monomer (black) and dimer (red) of the protein. (D) Protein profile of purified preparations of DIIIE1wt and the seventy-four Ala-mutants, as indicated, after renaturation by dilution and Ni-NTA chromatography using Imidazol 50mmol/L as elution condition. All bacterial cultures were grown to a similar OD and protein samples were diluted to the same volume required for each step of the purification procedure. (E) Yield of the purification of DIIIE1wt and the seventy-four Ala-mutants. A color code is used to highlight the mutants with very low yield values. (F) Representative chomatographic profiles of SEC obtained for protein refolding (blue) and re-chromatography of peak c (pink) for DIIIE1wt and SDS-PAGE analysis of the collected fractions, as indicated. (G) Circular dichroism spectra analysis of the purified preparation of DIIIE1wt. (H) Chromatograms of refolding by SEC of representative Ala-mutants. Chromatograms and the corresponding mutant protein are color coded. (I) Representative protein profile of the purified preparation of Ala-mutants after refolding by SEC and re-chromatography.

## Description

The four serotypes of dengue virus (DENV1-4) are arthropod-borne pathogens that infect 390 million people yearly in the tropical and subtropical regions worldwide and cause a potentially fatal disease (Silva et al., 2020) . DENV1-4 are enveloped viruses with three structural proteins namely capsid, membrane and envelope (E) protein (Nanaware et al., 2021) . The E protein covers most of the surface of the virion and mediates early events of the infection like virus entry to cells and membrane fusion to release the viral RNA to the cytoplasm (De La Guardia & Lleonart, 2014) .

There are 90 copies of the E protein homodimer in the surface of DENV particles. Monomers of the E protein are folded in three structural domains I (DI), II (DII) and III (DIII) (Fahimi et al., 2018) . The DIII is the most protuberant region in the virion and exhibits the highest sequence variability in the E protein. The DIII is involved in the interaction with soluble factors of innate immune response in human plasma, with cellular receptors, and is target of potent neutralizing antibodies (Beltramello et al., 2010; Huerta et al., 2016) . Recombinant DIII proteins has been evaluated as antigens for sub-unit vaccines (Suzarte et al., 2014, 2015) and in the diagnostic of DENV infection (Nguyen et al., 2019) . Thus, the identification of residues and sequence stretches in the DIII that determine the interaction with human proteins provides pivotal information to understand virus adaptation through protein:protein interaction with its host.

The use of single-residue mutants obtained by alanine substitution of selected residues has proven to be useful in studies aimed at determining structure-function relationship (Chatellier et al., 1995) . This type of mutation introduces minor or non-perceptible changes in the structure of the protein while changing the characteristics of the side chain of the residue in the position that is mutated (Gray et al., 2017) . Here we describe a time-efficient experimental workflow used in the expression and purification of seventy-five ala mutants of the DIII of DENV1 that result in high yields of correctly folded proteins useful for protein interaction studies.

Recombinant protein construct corresponding to wild type DIII of the dengue virus serotype 1 PRS288690 (DIIIE1wt) comprises residues 289-399 of the E protein (GenBank: AAN32775.1); it includes residues of the linker between DI and DIII (289-309) in the structure of the E protein that allowed taking advantage of methionine 289 as a start codon of the protein constructs. DIIIE1wt also bears a hexa-histidine tag at the C-terminus to facilitate the purification process (Huerta et al., 2014) . The criterion used to select residues to be mutated to alanine was a relative accessibility higher than 15% and a ΔΔG lower than 4 kcal/mol (Chinea et al., 2015) .

Considering that the interest of studying the role of all surface-exposed residues of the DIII of the E protein comprises the purification of seventy-five proteins, the implementation of a time-efficient expression and purification protocol is a worthwhile effort. Consequently, all steps from the bacterial culture and protein purification were conceived and performed for 8 to 10 mutants in parallel. The average yield of cell biomass per liter of culture was 20 ± 2 g. This result is similar to a previous reports of high-yield expression (22.8 g/L and 17.3 g/L) of recombinant DIII in *E. coli* for a protein spanning residues 300-400 of the E protein of DENV3 and 4, respectively (Tripathi et al., 2008, 2011) . Thus, the inclusion of the DI-DIII segment has no apparent impact on protein expression within *E. coli* , neither toxicity on cell biomass development.

Recombinant DIIIs represented roughly 15% of the total protein content after cell disruption homogenate (see lane DIIIE1wt in Figure 1A ). Only one mutant protein corresponding to substitution of residue K310 for Ala showed lower representation in the total protein content analysis followed by SDS-PAGE ( Figure 1A ). The high degree of residue conservation of K310 (100%) along the four DENV serotypes is, per se, indicative of a crucial structural or functional role of this residue in the DIII. In fact, Pitcher et al reported that the mutation of K310 for Ala impeded the production of new virus DENV2 particles which was attributed to a critical role in inter-domain interactions in the folding of the E protein (Pitcher et al., 2015) . Our current result is indicative of a more local effect at the level of the DIII of the E protein.

DIIIE1wt and seventy-four mutant proteins were obtained as inclusion bodies through the heterologous expression in *E. coli* BL21 ( Figure 1B ). Washing of inclusion bodies with mild denaturing conditions (1% triton X-100, 1 M urea) made a significant contribution to purification of DIIIE proteins from protein contaminants ( Figure 1B ). Inclusion bodies of DIIIE1wt and mutants were solubilized quantitatively using GuHCl 6 M yielding 1,3-1,5 mg of total protein content per milliliter of bacterial culture.

After solubilization, refolding is the step that defines the pace and yield of the purification process (Singh & Panda, 2005) . Refolding by dilution followed by purification is generally preferable since aggregates of misfolded protein can be separated along with contaminants during subsequent purification steps. Solubilized inclusion bodies of DIIIE1 were subjected to refolding by dilution at different final concentrations from 100 to 20 µg/mL, and applied to a Ni-NTA chromatography. In this range of protein concentration there was not difference in the yield of refolding process or the results of the subsequent chromatography. Elution from Ni-NTA column was assayed with buffer containing 50, 100, 150 or 300 mM of imidazole ( Figure 1C ). As it is apparent from the SDS-PAGE analysis, eluting samples exhibit a major band migrating according to the expected position for DIIIE1 monomer, close to the molecular weight marker of 14 kDa ( Figure 1C, black arrow ). Although the yield of DIIIE1 monomer increases slightly with imidazole concentration during elution from Ni-NTA chromatography, at imidazole concentrations greater than 50 mM a significant fraction of DIIIE1 oligomers, dimers mainly ( Figure 1C, red arrow ), formed during protein refolding, coelutes with it. Therefore, 50 mM was established as the imidazole concentration for elution in Ni-NTA. The protein yield (0.8 mg per milliliter of bacterial culture) and protein band profile of the purified preparation of DIIIE1wt and seventy mutants was very similar ( Figure 1D ). A densitometric analysis of the SDS-PAGE protein profile resulted in an average purity of 91%. Aside for the major band corresponding to the protein monomer, small amounts of a dimeric form of the protein and high molecular weight aggregates were also observed ( Figure 1D ).

In addition to K310, three other mutant proteins were repeatedly obtained with lower yields after renaturalization by dilution followed by Ni-NTA chromatography: G304, D330 and P332 ( Figure 1E ). Different from K310, these three latter mutants were obtained at average levels of expression ( Figure 1A ). Similar to K310, residue P332 is strictly conserved in the DIII of the four DENV serotypes. In contrast, the positions of G304 and D330 show significant variability (Sukupolvi-Petty et al., 2007) indicating that, in the context of the E protein, considerable changes are tolerated in these two positions that do not significantly affect the formation of new virus particles. Of note, in the folded protein, residues G304, D330 and P332 are in close vicinity to the cysteine residues (C302 and C333) of the DIII. Together, these data suggest that mutations in residues G304 and D330 may find compensation in the context of the viral particle that are not present in the isolated recombinant DIII and that may influence the formation of the disulfide bond between the two Cys residues.

A major drawback of refolding by dilution is that small increments in the scale of the protein to be obtained lead to massive increments in refolding volume (Singh & Panda, 2005) . In consequence, processing time of refolding itself, and subsequent filtration and concentration steps become considerably time consuming, typically between 36 to 72 hours (Simonelli et al., 2013) . As an alternative to increment both, the scale and output of the purification of DIIIE proteins, we evaluated a purification procedure that consists on an initial affinity chromatography with Ni-NTA under denaturing conditions and subsequent refolding by size exclusion chromatography (SEC). Superdex 75 matrix was selected since it can resolve biomolecules within a range of molecular weight of 3000 to 70 000 Da. In this case the buffer exchange to eliminate denaturing conditions occur simultaneously to the separation of aggregates and, potentially, also the protein dimer. The SEC refolding process was evaluated in different conditions of pH and composition of column equilibration buffer, flowrate, as well as the amount and volume of protein loaded. The chromatographic- and protein profile by SDS-PAGE of DIIIE1wt was highly reproducible from 0,8 to 1.5 mL/min. However, it was significantly affected by the increment in initial protein concentration and the sample volume.

The chromatographic profile obtained for DIIIE1wt shows three main fractions ( Figure 1F ). The SDS-PAGE analysis of chromatographic fractions with elution volumes of 38 ml and 58 ml (peaks a and b in Figure 1F ), contain mainly high molecular weight aggregates and a dimeric form of the protein respectively. Peak c, in turn, show a major band close to the 14 kDa protein marker, corresponding to the expected molecular weight of the monomer of the protein. In this latter fraction it is noticeable the presence of the protein dimer although in ≤10% of total proteins, as estimated by densitometry. Purified preparations of recombinant proteins has been used as analytical tool in studies of protein:protein interactions. However, small amounts of a dimer or oligomers of the protein can greatly distort results in the characterization of affinity or stability of the interactions (Brand et al., 2017) . Moreover, a variable abundance of dimer and oligomer contaminants could be obtained for the different mutant proteins. Thus, a re-chromatography using the same SEC column (Superdex 75 HR 16/60) equilibrated in in sodium phosphate buffer (50 mM pH 6 and 150 mM NaCl) was introduced as a polishing step (peaks d in Figure 1F ).

The analysis of the secondary structure content of the final preparation showed a high coincidence between the estimate made from the circular dichroism spectrum and the experimental result obtained (RMSD: ~0.1%). Furthermore, the assignment carried out according to the crystallographic structure of DIIIE1 was similar to the value estimated from the spectral data ( Figure 1G ). The absence of α-helices and parallel β-sheets, in contrast to the presence of nearly 40% of antiparallel β-sheets, are elements that confirm the structural identity of the recombinant protein and its native conformation at the end of the purification.

The DIIIE1wt chromatographic profile was similar in the number of elution peaks and their relative abundance for most of the seventy-four mutants ( Figure 1H ). Consistent with previous observations in this work, the chromatographic profile of mutants G304, K310, D330 and P332, presented a higher proportion of high molecular weight oligomers. Final purified preparations of DIIIE1wt and mutants were obtained with an average yield of 0.8 mg of protein per 50 mL of bacterial culture and more than 95% of purity. For mutants G304, K310, D330 and P332 bacterial culture was scaled up to increase the starting material and repeated purification processes of the same scale were performed to obtain a similar protein amount of purified protein with high homogeneity ( Figure 1I ).

Purified DIIIE1wt and mutants were evaluated for the sequence integrity and the formation of the disulfide bond between the two cysteine residues by mass spectrometry, and compared with expected isotopical mass for the oxidized protein (Extended data). Observed mass values in each case corresponded to expected values, with deviations ≤0.005% of the mass. Only mutants L292, L294, M301, Q347 and N366 the deviations from expected mass were larger than 0.5 Da (-0.56, 0.51, -0.52, -0.6 and 0.87, respectively). However, these deviations do not correspond to the difference between a reduced and oxidized disulfide bond which is 2 Da (Tsai et al., 2013) .

The estimated total time for purification of a sample from cell culture to the have the sample ready for analysis is approximately 27 to 30 h, including an overnight time for bacterial culture (18 h), which reduces the researcher time to approximately 12 h, separated in 1.5 working days. In our hands, the process was manageable processing up to 10 mutants in parallel fitting to this timing.

In summary, we present a time efficient strategy to obtain seventy-five alanine-mutants of the DIII of the E protein of DENV, serotype 1 in highly homogeneous preparations suitable for the use in studies of protein:protein interaction. Also, we present evidences about amino acid residues G304, K310, D330 and P332 as important for the stability of the recombinant DIII proteins.

## Methods

**Expression and purification of recombinant proteins**

Cryopreserved aliquots of *E. coli* BL21 (DE3) clones containing the corresponding plasmid were used to inoculate 50 mL of ZYM50502 medium (Studier, 2005) supplemented with ampicillin at 100 µg/mL in a 1 L erlenmeyer flask, which was then incubated for 12 hours at 37°C, 300 r.p.m. The *E. coli* cellular pellet was harvested by centrifugation at 3500 r.p.m. during 40 min. Cell pellet was suspended with a politron in 15 mL of phosphate buffered saline: 8 mM Na _2_ HPO _4_ , 2 mM KH _2_ PO _4,_ 137 mM, NaCl, 2.7 mM KCl, pH 7.4 (PBS), 10 mM EDTA and 1 mM PMSF (Rupture buffer), and disrupted with 6 cycles of intermittent sonication on ice for 30 s with 30 s allowed for cooling. The homogenized fraction was centrifuged at 10000 r.p.m., 15 min and 4˚C and washed sequentially with 1% Triton X-100 and 1 M Urea, both diluted in 40 mL of Rupture buffer, and 6 mL of PBS. After centrifugation, the soluble fraction was discarded and washed pellet was solubilized with 6 M GuHCl, PBS pH 8.0, 300 mM NaCl and 10 mM Imidazole (Extraction buffer). Dissolved inclusion bodies were cleared by centrifugation at 10 000 r.p.m., 4ºC for 10 min. Final supernatant was stored at -20˚C until use.

**Refolding by dilution**

Solubilized inclusion bodies were delivered drop-wise to refolding buffer (PBS, 0.3 M NaCl, 50 mM imidazole) at ~1 ml/hour with stirring at 200 rpm and 4 °C, to obtain a final protein concentration of 0.02 mg/ml. Next, protein solution was filtrated through 0.2 mM using a Sartobrand P capsule (Sartorius, Germany). Protein solution was concentrated using a Sartocon slice cassette co/2 kDa (Sartorius, Germany).

**Ni-NTA affinity chromatography**

**Native conditions**

Protein preparation obtained by refolding by dilution was incubated with 0.3 mL of Ni-NTA-Agarose (Qiagen, Germany) in a rocking shaker for 1 hr at 25 ° C. After this period the resin was packed in a column, and washed with 5 mL of PBS, 0.3 M NaCl, 20 mM imidazole. Next, bound protein was eluted by means of application of 900 μL of the same buffer but containing imidazole at 50, 100, 150 or 300 mM. Eluted protein was desalted by filtration gel on Sephadex G-25 (Amersham Pharmacia Biotech AB, Sweden) equilibrated with PBS. The resulting preparation was stored at -20 °C until use.

**Denaturing conditions**

Five milliliters of Ni-NTA-Agarose (Qiagen, Germany) were packed in an XK-26 (GE Healthcare, USA) column. Afterwards, the column was equilibrated with 8 mM Na _2_ HPO _4_ , 2 mM KH _2_ PO _4_ , 300 mM NaCl, 10 mM imidazole, 4 M GuHCl, pH 7.4 (Equilibrium buffer: EqB) and connected in line with a UV detector at 280 nm. Solubilized inclusion bodies were diluted with 10 fold the sample volume with EqB, and applied at 1.0 mL/min in the Ni-NTA column. Next, the column was washed with 2 column volumes of EqB followed by same buffer but containing 20 mM imidazole, until absorbance returned to baseline. Bound protein was eluted with EqB containing 100 mM imidazole. Protein fractions were collected, characterized for protein composition and concentration. Selected fractions were stored at -20˚C until use.

**Size exclusion chromatography**

SEC was performed using a Superdex 75 ^TM^ 16/600 Prep grade column (GE Healthcare, USA). Protein was operated at 25 ⁰C, connected in line with a UV detector set at 226 nm. For protein refolding, the column was equilibrated with 50 mM Na _2_ HPO _4_ , 150 mM NaCl, pH 7.4. Protein samples were applied at a flowrate of 0.8 mL/min. Protein aggregates were removed by injection of 3 mL of 8 M urea after each chromatographic run. The column was thoroughly cleaned with 60 mL of 0.5 M NaOH after four refolding processes.

When SEC was used as the final polish chromatography, the column was equilibrated with 50 mM Na _2_ HPO _4_ , 150 mM NaCl, pH 6.0 and protein samples were applied at 1,5 ml/min. In both chromatography runs, protein fractions with the highest monomer proportion, as evaluated by SDS-PAGE, were concentrated by ultrafiltration and stored at -20ºC until used.

**Protein quantitation**

Samples were quantified using a bicinchoninic acid protein assay kit (Pierce, USA). Protein samples collected from SEC were quantified by their absorbance at 280 nm, using a NanoPhotometer NP80 (Implen GmbH). The extinction coefficient was calculated using the ExPASy-ProtParam tool ( https://www.expasy.org/resources/protparam ).

**Denaturing polyacrylamide gel electrophoresis (SDS-PAGE)**

Proteins were separated in minigels using the standard Laemmli procedure for discontinuous gel electrophoresis under denaturing conditions (Gallagher, 2006; Laemmli, 1970) . Gels were prepared at 12.5 % T and 3% C in 0.37 M Tris- HCl, 0.1% SDS, pH 8.8 and the concentrator gel was used at 6 % T, 3% C in 0.125 M Tris-HCl, 0.1% SDS, pH 6.8. The samples were diluted in sample buffer 0.063 M Tris-HCl, 1% SDS, 12.5% glycerol, 0.001% bromophenol blue, 2.5% β-mercaptoethanol (for analysis under reducing conditions), pH 6.8, and heated for 2 min at 95 ^o^ C. The electrophoretic run was performed at 20 mA/gel in 0.025 M Tris-HCl electrode buffer, 0.192 M glycine, 0.1% SDS, pH 8.3. After the electrophoretic run, protein bands were visualized with Comassie blue staining (Sasse & Gallagher, 2003) and silver staining (Rabilloud et al., 1994) .

**Circular dichroism**

A sample of purified DIIIE1wt was diluted to 25 μM in 10 mM sodium phosphate buffer pH 6 and 30 mM NaCl. The far-UV DC spectrum (200–250 nm) was obtained on the J-1500 dichograph (Jasco, Japan) at 24 °C. For the estimation of the secondary structure content from the circular dichroism spectrum and the assignment from the three-dimensional structure (pdb file: 3IRC), the BeStSel Method was used (Micsonai et al., 2015) . Data processing was carried out using the server with the same name as the method ( http://bestsel.elte.hu/ssfrompdb.php ).

**Analysis by MS**

Mass spectra were acquired with a hybrid octagonal geometry QTOF-2TM mass spectrometer (Micromass, UK) fitted with a Z-spray electrospray ionization source. The software package MassLynx, ver. 3.5 (Waters, USA) was used for spectra acquisition and processing.

Aliquots of 20 μl containing 4-40 μg of of DIIIE1wt and mutants were desalted in ZipTip C-18 columns (Millipore, USA), following the manufacturer´s instructions. The elution was carried with 3 μl of water/acetonitrile/formic acid (60 and 0.1% respectively). The samples were applied in the borosilicate capillary covered with gold (Micromass, UK), for the analysis in the spectrometer nanospray source, using 900 V and 35 V for the capillary and the entry cone. The spectrometer was calibrated with the saline solution compose of sodium iodides and cesium in the 50-2000 m/z range.

Spectra were analyzed in MassLynx 4.0 for the maximum entropy method, using the 12000:14000 Da range with 1.00 Da/canal and Gaussian model with 0.75 Da of wide by middle height, and minimum intensity threshold of 45% for adjacent peaks. Calculation was executed as this program implement.

## Extended Data

Description: Characterization of purified preparations of Ala-mutants of the recombinant protein DIIIE1 using ESI-MS. Resource Type: Dataset. DOI: 10.22002/3be4v-35679
